# A Comparison of Dietary and Caloric Restriction Models on Body Composition, Physical Performance, and Metabolic Health in Young Mice

**DOI:** 10.3390/nu11020350

**Published:** 2019-02-07

**Authors:** Nicholas J. G. Smith, Jade L. Caldwell, Marie van der Merwe, Sunita Sharma, Matthew Butawan, Melissa Puppa, Richard J. Bloomer

**Affiliations:** School of Health Studies, University of Memphis, Memphis, TN 38152, USA; njsmith@stetson.edu (N.J.G.S.); jlcldwl2@memphis.edu (J.L.C.); mvndrmrw@memphis.edu (M.v.d.M.); suneeta.sharma44@gmail.com (S.S.); mbbtawan@memphis.edu (M.B.); mpuppa@memphis.edu (M.P.)

**Keywords:** diet, fasting, exercise, insulin, body composition

## Abstract

Time-restricted feeding (TRF), alternate day fasting (ADF), and the dietary restriction model known as the Daniel Fast (DF; a vegan/non-processed food diet plan) have garnered attention recently as nutritional interventions to combat obesity. We compared the effects of various dietary models on body composition, physical performance, and metabolic health in C57BL/6 mice. Sixty young C57BL/6 male mice were assigned a diet of TRF, ADF, DF, caloric restriction (CR), a high-fat Western diet (HF) fed *ad libitum*, or standard rodent chow for eight weeks. Their body composition, run time to exhaustion, fasting glucose, insulin, and glucose tolerance test area under the glucose curve (AUC) were determined. Compared to the HF group, all groups displayed significantly less weight and fat mass gain, as well as non-significant changes in fat-free mass. Additionally, although not statistically significant, all groups displayed greater run time to exhaustion relative to the HF group. Compared to the HF group, all groups demonstrated significantly lower fasting glucose, insulin, and Homeostatic Model Assessment of Insulin Resistance (HOMA-IR), as well as improved glucose tolerance, and the ADF group displayed the best fasting glucose and glucose tolerance results, with DF having the best HOMA-IR. All investigated fasting protocols may improve body composition, measures of insulin sensitivity, and physical performance compared to a high-fat Western diet. The DF and ADF protocols are most favorable with regards to insulin sensitivity and glucose tolerance. Since our selected dietary protocols have also been investigated in humans with success, it is plausible to consider that these dietary models could prove beneficial to men and women seeking improved body composition and metabolic health.

## 1. Introduction

Recent estimates indicate that 36.5% of adult Americans are classified as obese, defined as a body mass index (BMI) of ≥30 kg∙m^−2^ [1]. Obesity is associated with negative alterations to measures of overall health, including increased fat mass (FM), abdominal FM, a higher ratio of FM to fat-free mass (FFM) [2], decreased physical fitness [3], and in certain cases, compromised glucose homeostasis. Given that the prevalence of obesity is expected to increase in the coming decades, it is clear that novel interventions are needed to favorably impact body composition and physical fitness in obese individuals. Moreover, because obesity is strongly linked to insulin resistance [4], developing strategies to reduce body weight is important with regards to metabolic health and reducing the risk of type 2 diabetes.

In recent years, three dietary modifications have been increasingly promoted as potential aids in preventing and treating obesity and obesity-related dysfunction: time-restricted feeding (TRF), alternate day fasting (ADF), and the dietary restriction model known as the Daniel Fast (DF). TRF, or intermittent fasting, restricts daily feeding periods to designated hours of the day (e.g., only eating from 12:00 p.m.–7:00 p.m.), extending the typical overnight fast by several hours. Research in both humans [5,6] and animals [7,8,9] has indicated that TRF may be a successful method for reducing body mass and FM, while maintaining FFM [5,6]. Physical fitness levels are also maintained in normal weight individuals following short-term TRF [5,10]. Similar results have also been reported in other TRF models (C57BL/6J mice and *Drosophila*) relative to control groups [11,12]. Additional human [13,14,15] and animal [9,16,17] TRF studies have noted benefits with regards to glucose control.

Traditional ADF protocols involve repeated cycling between 24-h periods of *ad libitum* caloric intake and 24-h periods of fasting. Recent work has also utilized a modified ADF, which permits the consumption of small meals on fast days, typically consisting of 25–30% of normal daily caloric consumption [18,19]. ADF has been shown to improve measures of body composition in humans, resulting in significant reductions in FM [20,21,22,23,24,25], visceral FM [23], and waist circumference [23,26], while maintaining FFM [19,20,24]. To our knowledge, no studies have reported measuring physical fitness following ADF. ADF in humans has been shown to decrease fasting glucose [19] and insulin [22], and to improve HOMA-IR [19,27], while animal studies have noted improvements in insulin sensitivity [28].

The DF is a religiously motivated fast, derived from the biblical Book of Daniel [26]. Individuals participating in a DF may consume fruits, vegetables, whole grains, nuts, legumes, seeds, and healthy oils (such as olive oil) ad libitum. Products that contain additives, preservatives, processed foods, as well as caffeine and alcohol, are prohibited. The DF has been shown to result in an average weight loss of 5–6 pounds within three weeks, with noted favorable changes in body composition in both humans [26,29,30,31] and animals [32]. While no DF study to date has examined physical performance in humans, our recent animal study noted a significant increase in exercise capacity (run time to exhaustion) in rats consuming a DF diet and participating in regular exercise, relative to control groups consuming a Western diet [32]. With regard to glucose homeostasis, studies in both humans [26,30,31,33,34,35] and animals [36] have noted positive findings when incorporating the DF plan.

When considering the available literature, it appears that all three plans—TRF, ADF, and DF—could prove beneficial in combatting obesity and related variables. However, it is uncertain as to whether or not one plan is better than another with respect to selected variables. To our knowledge, no study has compared the above dietary interventions within one protocol, inclusive of outcomes related to body composition, physical performance, and metabolic health. The present study aimed to accomplish this goal, while also including a commonly utilized caloric restriction (CR) model and a standard chow diet within the research design. Specifically, a group of mice consuming only chow served as a “healthy” control, while the CR condition was used for comparison to our more novel interventions, as this CR has been studied extensively for the weight loss and metabolic health benefits.

## 2. Materials and Methods

### 2.1. Mice and Interventions

Four-week-old C57BL/6 male mice (*n* = 60) were purchased (Envigo, Pratville, AL, USA) and housed in a U.S. Food and Drug Administration (FDA)-approved facility on the University of Memphis campus. Animals were randomly assigned to a specific intervention or control group. Mice were co-housed (2–3 mice per cage) and entrained to a reverse 12-h light:12 h dark cycle for 2 weeks, with lights off between the hours of 7:00–19:00, as done previously [37]. During the entrainment period, mice had ad libitum access to an unpurified standard rodent chow (Chow, Teklad Global 2018, Envigo Laboratories Inc.; 18% fat, 58% carbohydrates, 24% protein; 3.1 kcal/g).

Following entrainment, eight mice were maintained on the chow diet (CHOW) for the entire study and served as a healthy control group. The remaining mice (*n* = 52) entered a lead-in period where they were fed a 45% high-fat diet (HF, D12451, Research Diets, Inc., New Brunswick, NJ, USA; 45% fat (predominantly lard), 35% carbohydrate, and 20% protein; 4.73 kcal/g) for six weeks to induce weight gain. Following the lead-in period, the intervention was initiated for six groups with different dietary profiles (see Table 1, Table 2 and Table 3 for dietary composition of each diet). The sample size (*n* = 8–9 per group that completed the study) was based on our prior work using a very similar design [37], considering the outcome measures included in this present study. The small discrepancy in sample size between groups (i.e., 8 or 9 animals) was a request from the attending veterinarian, as initial concern existed regarding the somewhat restrictive nature of the DF, TRF, and ADF plans; our plan was to have one additional animal in these groups in the event of animal loss.

Group 1 (HF, *n* = 8) continued to have ad libitum access to the 45% high-fat diet.

Group 2 (sCHOW, *n* = 8) were switched back to ad libitum access to standard rodent chow.

Group 3 (DF, *n* = 9) had ad libitum access to a purified, high-fiber, vegan-based diet. The DF diet consumed by Group 3 (Research Diets; product: D13092801) was custom-made by Research Diets, Inc., was used in our prior rodent study [32], and is based on the average macronutrient sources and quantities of the dietary intakes of human participants following the Daniel Fast in our previous studies.

Group 4 (CR, *n* = 8) received the 45% high-fat diet at 80% of ad libitum intake, as determined during week six of the lead-in period. This 20% reduction is in line with many human-based hypocaloric weight loss diet programs, as well as animal studies involving CR.

Group 5 (TRF, *n* = 9) had ad libitum access to the 45% high-fat diet for 6 h during the first half of their active phase (7:00–13:00).

Group 6 (ADF, *n* = 9) had ad libitum access to the 45% high-fat diet every other day. That is, on day 1 they received as much food as desired during the entire 24-h period. On day 2, they received no food. On day 3, they received ad libitum access to food, and so on. It should be understood that our approach represents a more stringent approach, as compared to the often-used “very low calorie” ADF protocol adopted in some human studies, in which “fast days” allow a small number of calories (e.g., 500) to be consumed during the day.

The mice remained on their particular diets for seven weeks, and then post-testing began. Post-testing was initiated by treadmill run-time-to-exhaustion testing. Due to equipment and time constraints, run-time-to-exhaustion testing was conducted over a two-day period for each cohort of 30 animals. For example, twelve mice completed run-time-to-exhaustion testing on the last day of the seventh week, and 18 mice completed run-time-to-exhaustion testing on the first day of the eighth week. With the exception of the four ADF mice, which all ran on their feed days, two mice from each of the intervention groups completed testing on each testing day. This was done to ensure that intervention groups were split up evenly between the two days in an effort to control potential confounding factors. Five days after run-time-to-exhaustion testing, glucose tolerance testing was conducted to ensure that glucose tolerance was not impacted by the acute exercise. Body composition was measured on days one and two of week nine for ADF mice, to ensure that ADF mice were scanned on both fed and fasting days. Mice were euthanized on days two and four of week nine.

All mice continued on their diets until all testing was completed (~middle of week 9). Water was provided ad libitum throughout the study period. The amount of food consumed was measured daily, and the weights of the mice were taken on alternating days. Following the conclusion of post-testing, mice were euthanized via cervical dislocation (using isoflurane inhalation for anesthesia).

### 2.2. Measurement of Body Composition

Animals underwent magnetic resonance imaging (MRI) scans for the determination of body mass/body fat. This was done during the sixth week of the lead-in period (baseline) and the ninth intervention week (post-intervention) using a small animal MRI unit (EchoMRI™, Houston, TX, USA), which uses a specialized NMR-MRI-based technology to rapidly measure lean and fatty tissue in small animals. Baseline and post-intervention scans were performed on the same days for all animals, during the last hour of the animal’s inactive (light) phase. With regard to the ADF group, baseline and post-intervention scans were performed following 24 h of feeding.

### 2.3. Measurement of Physical Fitness

Animals underwent a treadmill run-time-to-exhaustion test using a motorized treadmill (EXER 3/6; 1055-SRM; Columbus Instruments, Columbus, OH, USA) with a 5% incline. Animals ran at 20 m/min for 30 min and 25 m/min for the remaining time, until they reached exhaustion. A warm-up was provided for 15 min (5 min at 5 m/min, 5 min at 10 m/min, and 5 min at 15 m/min). Exhaustion was defined as the time at which mice were no longer able to continue running and sat on the shock grid with all four paws on the grid for 5 s, despite gentle hand prodding. The very mild electric shock was only used when mice did not respond well to gentle hand prodding and at the end of the run-time-to-exhaustion test, to determine the stopping point. The frequency and amplitude of shock was as low as possible (3 Hz) to motivate the animals to remain on the treadmill belt, without causing unnecessary distress.

The run-time-to-exhaustion testing was conducted twice, once prior to starting the intervention period (during the sixth week of the lead-in period, as a familiarization trial) and once at the end of the eighth intervention week. The first run-time-to-exhaustion test was used to acclimate the mice to the treadmill and the run-time-to-exhaustion protocol. Data are not available for this test. The second test was used as a primary dependent variable, to characterize the physical capacity of each group after the eight-week intervention. All run-time-to-exhaustion tests were conducted in the dark approximately 2 h after the initiation of the active phase (lights off). Run-time-to-exhaustion tests were conducted on days that ADF mice had access to food (i.e., fed days).

### 2.4. Glucose Tolerance Test Protocol

A glucose tolerance test (GTT) was performed following the lead-in (pre-intervention) and intervention periods (post-intervention). All mice were fasted eight hours prior to pre and post-intervention testing, except TRF mice at post-intervention. Due to TRF protocol, on the day of post-intervention testing, animals in the TRF group had been fasting since 2:00 p.m. the day prior. This fasting period was standard for their dietary protocol. Post-testing occurred after a feeding day for ADF mice to ensure equal hours of fasting. Blood was collected via the tail vein. Fasting glucose levels were measured using a standard glucometer (OneTouch^®^, Ultra). Mice then received 1 g glucose/kg total bodyweight via intraperitoneal injection, and blood (~10 ul) was collected at 30, 60, and 90 min after injection to measure glucose clearance. These measurements were used to calculate the following variables: fasting glucose and area under the glucose curve (AUC).

### 2.5. Glucose Homeostasis

For fasting insulin and glucose levels, blood was collected from the sub-mandibular vein after an eight-hour fast. At the end of the lead-in period, blood was collected from all CHOW mice and a subgroup of animals (*n* = 20) consuming the HF diet (this group did not overlap with the GTT subgroup). These measurements were used to calculate the changes in fasting glucose and insulin induced by the six-week HF diet consumption. A post-intervention facial bleed was performed on all mice immediately prior euthanasia. All mice were fasted, as previously mentioned, and euthanized at the beginning of their active phase. HOMA-IR was calculated as previously described, using the equation: (Plasma Glucose [mg/dL] × Plasma Insulin [ng/mL])/2658 [38].

### 2.6. Biochemical Analyses

Glucose samples other than those analyzed via glucometer, as well as triglycerides and total cholesterol, were analyzed using a Vet Axcel clinical chemistry system (Alfa Wasserman, Diagnostic Technologies LLC, West Caldwell, NJ, USA). Fasting insulin was analyzed using a Mouse Ultrasensitive Insulin ELISA kit (Alpco, Salem, NH, USA) using a PowerWave 340 microplate reader (BioTek, Winooski, VT, USA). Samples were analyzed in duplicate.

### 2.7. Data Analyses

Prior to analysis, we tested data for normality using the Shapiro–Wilk normality test. If data were not normally distributed, the non-parametric Kruskal–Wallis test with Dunn’s multiple comparison was performed. For all data that were normally distributed, a one-way analysis of variance was used, with Tukey post hoc testing using Prism 7 (GraphPad Software, San Diego, CA, USA). Single degree-of-freedom contrasts were used to further compare groups. Although the CHOW condition was included as a “healthy” control, this group was included in the model to assess both the potential positive and negative outcomes of the various dietary interventions. An alpha value of 0.05 was used for all statistical testing. Cohen’s *d* was calculated for the run time to exhaustion data using Microsoft Excel (Microsoft, Redmond, WA, USA), in order to further explore the differences observed between groups. Effect sizes were interpreted according to Cohen’s guidelines (i.e., *d* = 0.2 represents a small effect size; 0.5 represents a medium effect size; and 0.8 represents a large effect size) [39]. Data are presented as mean ± SEM.

## 3. Results

### 3.1. Overview

Three animals died during the course of the study. One animal died during the sixth week of the lead-in period, due to sepsis caused by an injury of unknown origin sustained to the left hind limb (this animal was initially unassigned). Two animals (one from the CHOW group and one from the DF group) died during the sixth week of the lead-in period while performing the run-time-to-exhaustion test. Both mice died instantly from injuries sustained as a result of falling between the shock grid and the treadmill belt. All remaining animals completed the 16-week study.

While multiple outcome variables are included in this study and presented below, we focus on the weight loss and body composition data as the primary outcome measures. Metabolic and physical performance data compliment this and are presented and discussed below.

### 3.2. Caloric Consumption Data

**Figure 1:**
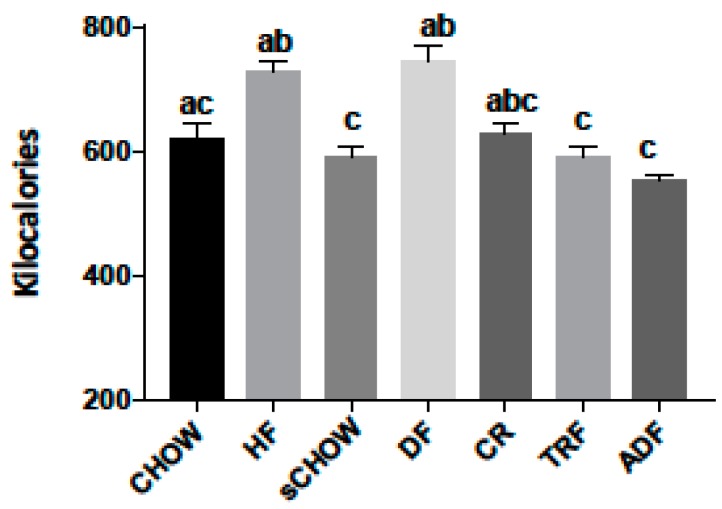
Total kilocalories consumed during the eight-week intervention period by male mice assigned to seven different dietary protocols. Values are mean ± SEM, *n* = 7–9 mice per group, using the Kruskal–Wallis test. Bars without a common letter differed. HF and DF > ADF (*p* < 0.001). HF > sCHOW (*p* = 0.010). HF > TRF (*p* = 0.009). DF > sCHOW (*p* = 0.016). DF > TRF (*p* = 0.014).

Caloric consumption during the eight-week intervention period (Figure 1) demonstrated that there was no significant difference between the CHOW, HF, DF, and CR groups (*p* > 0.050). The total caloric consumption was significantly greater for the HF group versus ADF (*p* < 0.001), sCHOW (*p* = 0.010), and TRF (*p* = 0.009). The total caloric consumption of the DF group was also significantly greater than the ADF (*p* < 0.001), sCHOW (*p* = 0.016), and TRF (*p* = 0.014) groups.

### 3.3. Anthropometric Data

**Figure 2:**
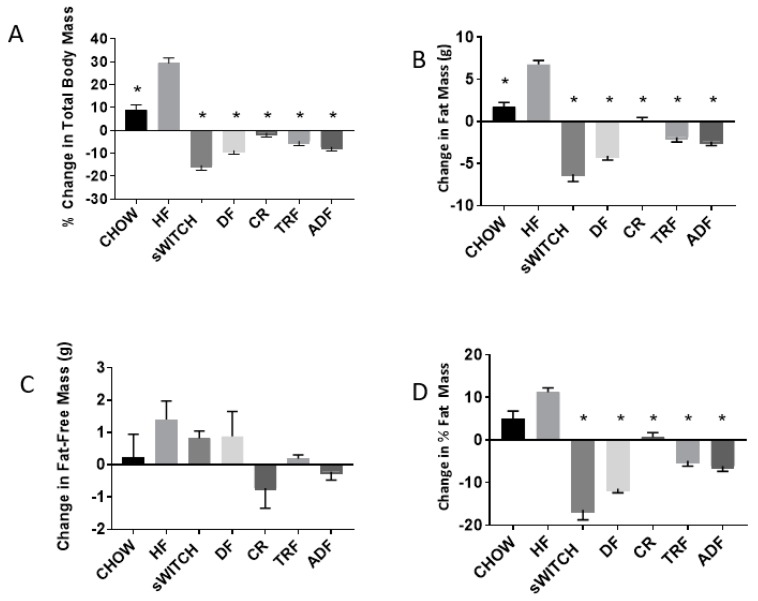
Percent change in total body mass (**A**), fat mass (**B**), fat-free mass (**C**), and percent fat mass (**D**) of male C57BL/6 mice assigned to different dietary protocols for eight weeks. Values are changed post-intervention as compared to pre-intervention. Values are mean ± SEM, *n* = 7–9 mice per group, In (**A**), * indicates a group effect for change in body mass: HF different from all groups (*p* < 0.001); in (**B**), * indicates a group effect for change in FM: HF different from all groups (*p* < 0.001); in (**C**), no group effect was observed (*p* > 0.050); and in (**D**), * indicates a group effect for change in % FM: HF different from sCHOW, DF, CR, TRF, and ADF (*p* < 0.001).

All anthropometric data are presented in Table 4 and Figure 2. The total body mass of HF and CHOW increased from baseline to post-intervention, while the total body mass of sCHOW, DF, CR, TRF, and ADF decreased from baseline to post-intervention. Several group effects were noted. HF gained significantly more body mass than all other groups (*p* < 0.001). CHOW gained significantly more body mass than CR (*p* < 0.001), DF (*p* < 0.001), TRF (*p* = 0.003), and ADF (*p* < 0.001). Additionally, the decrease in body mass noted for sCHOW was significantly greater than that of CR (*p* < 0.001), TRF (*p* = 0.003), and ADF (*p* = 0.034).

The sCHOW, DF, TRF, and ADF groups displayed decreased FM after the eight-week intervention, while the CHOW, HF, and CR groups displayed an increase in FM. The HF group gained more FM (6.76 ± 0.46 g) than all other groups (*p* < 0.001). The greatest decrease in FM was observed in the sCHOW group, which lost significantly more FM (6.275 ± 0.86 g; *p* < 0.005) than all other groups, with the exception of DF (−4.10 ± 0.49 g; *p* = 0.119). The change in FM for CHOW was significantly different from sCHOW (*p* < 0.001), DF (*p* < 0.001), TRF (*p* < 0.001), and ADF (*p* < 0.001). Change in FM for DF was significantly different from CR (*p* < 0.001). No significant differences were observed with regard to change in FM between the DF, TRF (1.89 ± 0.55 g), and ADF (2.39 ± 0.49 g) groups (all *p* > 0.050).

The change in percent FM from initiation of intervention to the final day of the intervention displayed several significant group effects. The increase in percent FM observed in HF was significantly greater than that of sCHOW, DF, CR, TRF, and ADF (*p* < 0.001). The increase in percent FM seen in CHOW was significantly greater than that of sCHOW (*p* < 0.001), DF (*p* < 0.001), TRF (*p* < 0.001), and ADF (*p* < 0.001). The decrease in percent FM for sCHOW was significantly greater than for CR (*p* < 0.001), TRF (*p* < 0.001), and ADF (*p* < 0.001). The decrease in percent FM noted for DF was significantly greater than that of CR (*p* < 0.001) and TRF (*p* = 0.031). The increase in percent FM of CR was significantly greater than that of ADF (*p* = 0.021).

No main effect or group effect was noted for the change in FFM from baseline to post-intervention. However, the change in FFM between HF (which gained FFM) and CR (which lost FFM) displayed a tendency towards significance (*p* = 0.068).

### 3.4. Run-Time-To-Exhaustion Data

Data for the run time to exhaustion are presented in Table 4. No significant effects were noted (*p* > 0.050). However, large effect sizes were observed when comparing DF to HF (*d* = 1.10; 95% CI: −2.746 to 0.539), TRF to HF (*d* = 0.99; 95% CI: −2.341 to 0.366), and ADF to HF (*d* = 1.10; 95% CI: −4.205 to 2.014).

### 3.5. Blood Lipids

With regards to triglycerides, no significant differences were observed (*p* > 0.050). Total cholesterol measurements (mg/dL) were as follows: 104 ± 2 (CHOW), 189 ± 8 (HF), 114 ± 4 (sCHOW), 104 ± 2 (DF), 142 ± 6 (CR), 118 ± 5 (TRF), and 151 ± 4 (ADF). The total cholesterol of the HF group was significantly greater than CHOW (*p* < 0.001), DF (*p* < 0.001), sCHOW (*p* = 0.006), and TRF (*p* = 0.018). Total cholesterol of the DF group was significant less than the CR (*p* = 0.021) and ADF (*p* = 0.004) groups. Total cholesterol of the CHOW group was significantly less than the ADF group (*p* = 0.012). No other significant comparisons with regard to total cholesterol were observed (*p* > 0.050).

### 3.6. Fasting Blood Glucose

**Figure 3:**
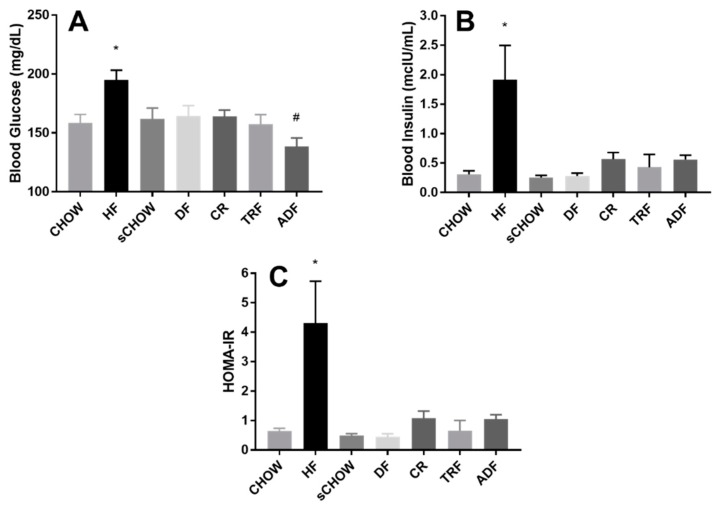
Post-intervention fasting blood glucose (**A**), fasting blood insulin (**B**), and HOMA-IR (**C**) of male C57BL/6 mice assigned to seven different dietary protocols for eight weeks. Values are mean ± SEM, *n* = 7–9 mice per group. (**A**) *A significant group effect noted: HF > all other groups (*p* < 0.050); # A significant group effect noted: ADF < sCHOW, DF, and CR (*p* < 0.050) (ANOVA); (**B**) *A significant difference noted: HF > Chow (*p* < 0.050), DF (*p* < 0.010), sChow, and TRF (*p* < 0.001); (**C**) *A significant difference noted: HF > sChow, DF, and TRF (*p* < 0.010) (Kruskal–Wallis).

With regard to pre-intervention fasting blood glucose levels, a significant group effect (*p* = 0.005) was noted when comparing the CHOW (169.1 ± 4.75 mg/dL) and HF (195.4 ± 4.75 mg/dL) groups. Post-intervention fasting blood glucose levels are presented in Figure 3A. A significant main effect of dietary intervention on fasting blood glucose was noted (*p* < 0.001). The fasting blood glucose of the HF group was significantly higher than all other groups (*p* < 0.050). The fasting blood glucose of the ADF group was significantly lower than for the sCHOW, DF, and CR groups (all *p* < 0.050).

### 3.7. Fasting Blood Insulin

A significant group effect (*p* = 0.067) was noted for pre-intervention fasting insulin when comparing the CHOW (0.19 ± 0.023 μIU/mL) and HF groups (0.867 ± 0.253 μIU/mL). Post-intervention fasting insulin levels are presented in Figure 3B. There was a significant increase in fasting insulin for HF vs. CHOW (*p* = 0.015), sCHOW (*p* < 0.001), DF (0.010) and TRF (*p* < 0.001).

### 3.8. HOMA-IR

Post-intervention HOMA-IR for each intervention group are presented in Figure 3C. Consistent with an increase in the fasting blood insulin levels, a significant increase HOMA-IR was calculated; HF > sCHOW (*p* = 0.005), DF (*p* = 0.001), and TRF (*p* = 0.001).

### 3.9. Glucose Area under the Curve

**Figure 4:**
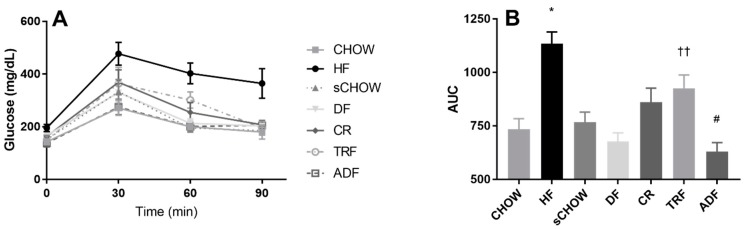
Post-intervention glucose tolerance test (**A**) area under the glucose curve (AUC) (**B**) of male C57BL/6 mice assigned to seven different dietary protocols for eight weeks. Values are mean ± SEM, *n* = 7–9 mice per group, ANOVA. (**B**) *A significant group effect noted: HF > CHOW, sCHOW, DF, CR, and ADF (*p* < 0.050); ^††^ A significant group effect noted: TRF > DF and ADF (*p* < 0.050); ^#^ A significant group effect noted: ADF < CR (*p* < 0.050).

A glucose tolerance test was performed post-intervention, and the area under the curve (AUC) was calculated for each intervention group (Figure 4A,B). A significant group effect was noted for the glucose AUC (*p* < 0.001). The glucose AUC of HF was significantly greater than that of all other groups (*p* < 0.050), with the exception of TRF (*p* = 0.087). The glucose AUC of TRF was significantly greater than DF (*p* = 0.012) and ADF (*p* = 0.002). The glucose AUC of CR was significantly greater than ADF (*p* = 0.038). Contrast tests revealed that the glucose AUC of DF was significantly lower than that of CR (*p* = 0.017) and TRF (*p* < 0.001).

## 4. Discussion

### 4.1. Anthropometric Findings

To our knowledge, this is the first study to compare the effects of dietary protocols mimicking caloric restriction, time-restricted feeding, alternate day fasting, and a purified vegan diet (i.e., the Daniel Fast) on measures of body composition, physical performance, and metabolic health in male C57BL/6 mice. Our data suggest that the consumption of a high fat diet under common feeding–fasting conditions such as CR, TRF, and ADF may protect against diet-induced obesity and associated metabolic and physical performance alterations. Mice fed a high fat diet ad libitum experienced significantly more overall weight gain than all other groups over the eight-week intervention period. This increase in body mass is characteristic of the growth of C57BL/6 mice given ad libitum access to a calorically dense diet [40,41]. For reference, the increase in body mass displayed by the CHOW group is characteristic of the normal growth of C57BL/6 mice [42]; whereas, the sCHOW group represents a return to normal growth following six weeks of high-fat feeding. It is important to note that no significant differences were observed with regard to the change in body mass between the CR, TRF, ADF, and even the DF groups.

With regards to body composition, the HF group gained significantly more fat mass than all other groups, resulting in a significant greater percent FM with an insignificant difference in FFM. Other groups consuming a high-fat diet under specified conditions saw no change (CR) to mild reductions in terms of fat mass (TRF and ADF). Interestingly, the DF group displayed the lowest post-intervention fat mass despite consuming the most kilocalories during the eight-week intervention period. This reduction in FM may have been attributed to the high omega-3 fatty acid [43] and fiber [44] consumption, as both have been theorized to reduce adiposity. Furthermore, animals in the DF group actually gained, albeit non-significantly, FFM along with the TRF group. This gain in FFM observed in the DF group is particularly notable because the purified vegan rodent diet consumed by the DF group contains only soy protein; it did not contain animal protein.

Many of our findings are consistent with the anthropometric measures reported by others utilizing DF, TRF, and ADF dietary protocols in animals. When considering both animal and human data, studies have consistently reported significant reductions in body mass following the DF [32], TRF [7,9,17,45], and ADF [25,46,47] protocols. The DF [32] and TRF [9,45] protocols have often resulted in reductions in FM, while only one animal study [48] has indicated that ADF results in decreased FM, relative to control. However, it should be noted that many of these studies, and all of the ADF studies mentioned here, use epididymal fat pad weight as an indicator of overall adiposity. The current study measured total FM using a non-invasive small animal MRI.

Prior data specific to the DF and FFM supports the data reported in the current study. Our previous work using male Long–Evans rats noted maintenance of FFM following 12 weeks of purified vegan rodent chow consumption, despite weight loss and decreased FM [32]. Measures of FFM have not been reported following TRF or ADF protocols in animals, but a number of human studies have reported reductions in body weight and FM while maintaining FFM following TRF [5,6] and ADF [19,20,24] protocols. It is evident from this prior work that these protocols can yield benefits in human subjects, which is supported by our present findings using animals.

Taken together, the data suggest that both nutrient restriction and time restricted feeding protocols are capable of producing favorable alterations in body composition. The DF protocol appears to produce the best results with regard to anthropometric measures of body composition in animal models, though more studies are needed to confirm these findings. Additionally, TRF and ADF protocols also appear to be viable options for individuals seeking to decrease body mass and FM while consuming a diet that is high in fat and simple sugar. However, more work is needed to determine additional health outcomes, as well as overall compliance to the various dietary regimens. This latter point is of great importance, as we can control all variables when conducting an animal study. The same is obviously not true when enrolling human subjects in a free living environment. Therefore, before we can draw firm conclusions as to which dietary regimen might be best, we need to have a better understanding of how individuals actually adhere to these plans long-term.

### 4.2. Run-Time-To-Exhaustion Findings

Considering the importance of physical activity to overall health and the changes to body composition with dieting, we sought to determine whether dietary patterns produced differences in physical performance between groups. No significant group effects were observed for the run-time-to-exhaustion test, likely due to large variation in run time and the small group size. Despite the lack of a significant effect, it is important to highlight the mean values for the main intervention groups. The mean run times for the DF (39.00 ± 2.90 min), TRF (40.00 ± 4.88 min), and ADF (27.78 ± 3.30 min) groups were greater than the mean run time for the HF group (16.38 ± 3.85 min). Additionally, large effect sizes were observed when the mean run time for the DF (*d* = 1.10; 95% CI: −2.746 to 0.539), TRF (*d* = 0.99; 95% CI: −2.341 to 0.366), and ADF (*d* = 1.10; 95% CI: −4.205 to 2.014) groups were compared with the HF group. These data indicate that the DF, TRF, and ADF protocols had a large potential effect on run-time-to-exhaustion performance, when compared with the HF group. This poorer performance by the HF group may be attributed to the relatively higher FM of the group, or possibly even contributed to by a diet-induced transition of exercising muscle fibers away from slow Type I fibers [49]. Furthermore, because the mean run time for the DF and TRF groups closely resembled the mean run time for the CHOW group (39.86 ± 7.87 min), the DF and TRF dietary appear to optimize performance relative to ADF. Future work is needed to more fully understand the impact of specific dietary regimens on physical performance.

Literature describing the effects of DF, TRF, and ADF on physical performance is scant. Only one known study has reported physical performance following a DF intervention in animals, and it demonstrated that the DF combined with regular exercise results in significantly greater aerobic performance than a high-fat diet combined with regular exercise [32]. However, run time between the DF and high-fat diet groups that did not participate in regular exercise did not differ significantly, findings that align well with the findings of the current study. With regard to TRF studies, one study [11] has reported that C57BL/6J mice following TRF protocols display significantly greater run time to exhaustion relative to a high-fat control, and another study demonstrated TRF improves physical performance (as measured by flight index) in *Drosophila* [12]. Additionally, multiple studies have demonstrated that TRF does not diminish physical performance in humans [5,10]. No animal model or human subject studies have reported any measurement of physical fitness following an ADF intervention. While the overall physiologic responses appear to be similar between humans and animals with regards to physical performance and adaptations, little has been done using either model. Clearly, more work is needed to investigate the impacts of DF, TRF, and ADF on measures of physical performance.

### 4.3. Metabolic Findings

Elevated blood glucose and impaired insulin sensitivity are hallmarks of metabolic syndrome and are thus a chief concern of individuals with diet-induced obesity. Our results demonstrate the negative effects of ad libitum consumption of a high-fat diet. These mice displayed elevated fasting blood glucose and insulin, resulting in a much greater HOMA-IR. Moreover, these mice experienced reduced glucose clearance, evidenced by the greater AUC following a GTT.

Interestingly, mice fed the same HF diet under various fasting routines experienced lower fasting blood glucose levels, with values of the ADF group being significantly lower than CR. A similar effect was noted for glucose clearance, as ADF AUC was significantly lower than CR—suggesting that ADF may be more impactful on lowering fasting blood glucose and could be an alternative dietary approach to CR, which has been previously suggested [21,50]. Considering that it has been anecdotally reported by some patients that complying to ADF is easier than daily CR, our results may further support ADF as an alternative to CR, for individuals who find it difficult to reduce caloric intake seven days per week as part of a lifestyle intervention. However, a recent human study demonstrated that ADF did not produce superior adherence [51].

It should also be mentioned that though the fasting insulin levels and HOMA-IR values did not differ between sCHOW, DF, CR, TRF, and ADF groups, the values for those fed the high-fat diet under fasting conditions were nearly double those of sCHOW and DF groups. These results suggest that specific alterations in dietary composition might be more beneficial than simply modifying the amount or timing of food consumption.

It is well known that accumulation of adipose tissue, specifically visceral, correlates with the development of insulin resistance [52,53]. It has been proposed that for every standard deviation increase in visceral adipose tissue, an 80% increase in risk of developing insulin resistance occurs [54]. This accumulation of visceral fat results in the secretion of adipokines and inflammatory cytokines that alter glucose homeostasis through the attenuation of insulin signaling [55,56]. In the current study, fat mass of all intervention groups was substantially lower than that of the HF group (14.72 ± 0.92 g), with the exception of the CR group (10.56 ± 0.62 g). Thus, the findings regarding glucose homeostasis presented here (i.e., greater fasting blood glucose, fasting blood insulin, HOMA-IR, and glucose AUC observed in HF group, relative to the intervention groups) support the notion that glucose homeostasis is linked with adiposity.

With regards to the above, our data indicate that the consumption of a high-fat Western diet, in particular when fed ad libitum, may contribute to an increase in fat mass relative to the consumption of purified vegan and standard chow diets in mice, thus contributing to an increase in insulin resistance. These findings have been well-documented in human subjects consuming high-fat diets. However, in the case of ADF, the lowest levels of fasting glucose and smallest glucose AUC were observed, while simultaneously having increased levels of fasting insulin, more similar to CR and TRF. These results indicate that ADF produces results allowing the body to efficiently clear glucose loads, but exposure to the high-fat diet increases insulin release, albeit not to the level of ad libitum exposure.

## 5. Conclusions

To our knowledge, this is the first study to compare the effects of dietary protocols mimicking caloric restriction, the Daniel Fast, time-restricted feeding, and alternate day fasting on measures of body composition, physical performance, and measures of metabolic health in male C57BL/6 mice. The findings presented here indicate that the Daniel Fast, time-restricted feeding, and alternate day fasting may be effective options for improving anthropometric measures, glucose homeostasis, and physical performance, when compared with ad libitum high-fat feeding. Of course, when considering human subjects, long-term compliance to the specific dietary program needs to be considered. Without strong adherence, little benefit may be expected. Future research using animal models and human participants are needed to more fully elucidate the mechanisms responsible for the improvements noted in response to the Daniel Fast, time-restricted feeding, and alternate day fasting. Specifically, research should focus on the specific nutrient composition and caloric consumption of each group and relate it to the noted changes. For example, in the present study, the Daniel Fast group consumed the greatest number of kilocalories during the intervention period, yet the Daniel Fast group also had the lowest FM. Future research should determine the causes of the noted changes and seek to address these results more specifically. While the present study involved an animal model, we believe that these findings would also have relevance to human subjects, as the TRF [5,6], ADF [19,20,22], and DF [26,29] dietary protocols have also been reported to prove beneficial in several human subject studies. Direct comparison studies of the various dietary plans in human subjects will help to provide clarification with regards to this assertion.

## Figures and Tables

**Table 1:** Macronutrient composition and caloric density of experimental diets.

	DF (Daniel Fast)	HF (High-Fat)	CHOW
Macronutrient	kcal%	kcal%	kcal%
Protein	15	20	24
Carbohydrate	59	35	58
Fat	25	45	18
kcal/gm	3.9	4.7	3.1

**Table 2:** Nutrient composition of Daniel Fast and high-fat diets.

	DF		HF	
Nutrient	gm	kcal	gm	kcal
Casein	0	0	200	800
Soy Protein	170	680	0	0
DL-Methionine	3	12	0	0
Corn Starch	0	0	72.8	291
Corn Starch-Hi Maize 260	533.5	2134	0	0
(70% Amylose and 30% Amylopectin)				
Maltodextrin	150	600	100	400
Sucrose	0	0	172.8	691
L-Cystine	0	0	3	12
Cellulose, BW200	100	0	50	0
Inulin	50	50	0	0
Soybean Oil			25	225
Lard	0	0	177.5	1598
Flaxseed Oil	71	639	0	0
Safflower Oil, High Oleic	59	531	0	0
Ethoxyquin	0.04	0	0	0
Dicalcium Phosphate			13	0
Mineral Mix S10001	35	0	10	40
Calcium Carbonate	4	0	5.5	0
Mineral Mix S10026			10	0
Vitamin Mix V10001	10	40	0	0
Choline Bitartrate	2	0	2	0
Ascorbic Acid Phosphate, 33% active	0.41	0	0	0
Potassium Citrate, 1 H2O	0	0	16.5	0
Cholesterol	0	0	0	0
FD&C Red Dye #40	0.05	0	0.05	0

**Table 3:** Nutrient composition of standard chow.

Micronutrients			Amino Acids		
Calcium	%	1	Aspartic Acid	%	1.4
Phosphorous	%	0.7	Glutamic Acid	%	3.4
Non-Phytate Phosphorous	%	0.4	Alanine	%	1.1
Sodium	%	0.2	Glycine	%	0.8
Potassium	%	0.6	Threonine	%	0.7
Chloride	%	0.4	Proline	%	1.6
Magnesium	%	0.3	Serine	%	1.1
Zinc	mg/kg	70	Leucine	%	1.8
Manganese	mg/kg	100	Isoleucine	%	0.8
Copper	mg/kg	15	Valine	%	0.9
Iodine	mg/kg	6	Phenylalanine	%	1
Iron	mg/kg	200	Tyrosine	%	0.6
Selenium	mg/kg	0.23	Methionine	%	0.4
**Vitamins**			Cysteine	%	0.3
Vitamin A	IU/g	15	Lysine	%	0.9
Vitamin D_3_	IU/g	1.5	Histidine	%	0.4
Vitamin E	IU/kg	110	Arginine	%	1
Vitamin K_3_ (menadione)	mg/kg	50	Tryptophan	%	0.2
Vitamin B_1_ (thiamin)	mg/kg	17	**Fatty Acids**		
Vitamin B_2_ (riboflavin)	mg/kg	15	C16:0 Palmitic	%	0.7
Niacin (nicotinic acid)	mg/kg	70	C18:0 Stearic	%	0.2
Vitamin B_6_ (pyridoxine)	mg/kg	18	C18:1ω9 Oleic	%	1.2
Pantothenic Acid	mg/kg	33	C18:2ω6 Linoleic	%	3.1
Vitamin B_12_ (cyanocobalamin)	mg/kg	0.08	C18:3ω3 Linolenic	%	0.3
Biotin	mg/kg	0.4	Total Saturated	%	0.9
Folate	mg/kg	4	Total Monounsaturated	%	1.3
Choline	mg/kg	1200	Total Polyunsaturated	%	3.4

**Table 4:** Anthropometric and run-time-to-exhaustion data for male mice assigned to seven different dietary protocols for eight weeks.

	Body Mass (g)		FM (g)		FFM (g)		% FM		Run Time to Exhaustion (min)
Intervention Group	PRE	POST	PRE	POST	PRE	POST	PRE	POST	POST
CHOW	25.49 ± 0.66	27.79 ± 1.03 ^§^	2.48 ± 0.38	4.21 ± 0.71 ^§^	21.05 ± 0.48	21.30 ± 0.39	9.6 ± 1.4	14.6 ± 2.3	39.86 ± 7.87
HF	30.54 ± 1.01	39.60 ± 1.71*	7.96 ± 0.72	14.72 ± 0.92 *	20.64 ± 0.37	22.03 ± 0.85	25.8 ± 1.6	37.0 ± 0.9	16.38 ± 3.85
sCHOW	33.90 ± 0.79	28.65 ± 0.83 ^¥^	10.71 ± 0.68	4.43 ± 0.89 ^¥^	21.20 ± 0.34	22.02 ± 0.34	31.4 ± 1.4	15.0 ± 2.5	42.13 ± 9.01
DF	31.83 ± 1.02	28.91 ± 0.5	7.82 ± 0.84	3.72 ± 0.44 ^†^	21.97 ± 0.57	22.85 ± 0.76	24.5 ± 1.9	12.8 ± 1.4	39.00 ± 9.4
CR	34.12 ± 1.05	33.69 ± 1.01	10.55 ± 0.82	10.56 ± 0.62	21.50 ± 0.30	20.78 ± 0.86	30.6 ± 1.6	31.3 ± 1.4	30.63 ± 5.38
TRF	30.03 ± 1.06	28.47 ± 0.63	7.80 ± 0.89	5.91 ± 0.51	20.31 ± 0.31	20.50 ± 0.26	25.4 ± 2.2	20.5 ± 1.5	40.00 ± 10.68
ADF	30.96 ± 0.56	28.64 ± 0.40	7.75 ± 0.63	5.36 ± 0.35	21.13 ± 0.23	20.91 ± 0.38	24.8 ± 1.6	18.7 ± 1.1	27.78 ± 3.30

Values are mean ± SEM. For Body Mass, ^§^ denotes a group effect noted for change in body mass: CHOW different from sCHOW, DF, TRF, and ADF (*p* < 0.005); * denotes a group effect for change in body mass: HF different from all groups (*p* < 0.001); and ^¥^ denotes a group effect for change in body mass: sCHOW different from CR, TRF, and ADF (*p* < 0.050). For fat mass (FM), § denotes a group effect for change in FM: CHOW different from sCHOW, DF, TRF, and ADF (*p* < 0.005); * denotes a group effect for change in FM: HF different from all groups (*p* < 0.001); ^¥^ denotes a group effect for change in FM: sCHOW different from CR, TRF, and ADF (*p* < 0.001); and ^†^ denotes a group effect for a change in FM: DF different from CR (*p* < 0.001). For fat-free mass (FFM): no time effect was observed (*p* > 0.050) and no group effect was observed (*p* > 0.050). For the percent FM, no time effect was observed (*p* > 0.050). For run time to exhaustion, no group effect was observed (*p* > 0.050).
